# The outcomes of hip resurfacing compared to standard primary total hip arthroplasty in Men

**DOI:** 10.1186/1471-2474-14-161

**Published:** 2013-05-08

**Authors:** Kimona Issa, Amanda Palich, Tiffany Tatevossian, Bhaveen H Kapadia, Qais Naziri, Michael A Mont

**Affiliations:** 1 Rubin Institute for Advanced Orthopedics Center for Joint Preservation and Replacement Sinai Hospital of Baltimore, 2401 West Belvedere Avenue, Baltimore, MD, 21215, USA

## Abstract

**Background:**

The purpose of this study was to evaluate the clinical and radiographic outcomes of hip resurfacing patients and compare them to standard primary total hip arthroplasty procedures performed during the same period of time.

**Methods:**

One hundred and fourteen consecutive men who had a mean age of 50 years (range, 20 to 85 years) and who had undergone 120 hip resurfacing arthroplasties between 2007 and 2009 were compared to 117 consecutive men (120 hips) who had undergone a standard total hip arthroplasty during the same time period. The mean follow-up was 42 months (range, 24 to 55 months) for both groups. Outcomes evaluated included implant survivorship, hip scores, activity levels, and complication rates.

**Results:**

In the resurfacing hip arthroplasty cohort, implant survivorship was 98% with two patients requiring a revision surgery one for femoral neck fracture and another for femoral head loosening. In comparison, implant survivorship was 99% in the standard total hip arthroplasty cohort, with 1 revision due to peri-prosthetic fracture which was successfully treated with a femoral component revision. In the resurfacing and standard hip arthroplasty cohorts, the mean post-operative Harris hip scores had improved to 96 and 94 points, respectively and were statistically similar. The resurfacing cohort had achieved a significantly higher mean post-operative University of California Activity Score (6.7 versus 5 points). There were no differences in the complication rates between the two cohorts.

**Conclusion:**

When patients meet the appropriate selection criteria in the hands of experienced and high-volume arthroplasty surgeons, hip resurfacing provides excellent results at short- to mid-term follow-up.

## Background

Metal-on-metal hip resurfacing systems offer an alternative treatment to standard total hip arthroplasty in patients who have end-stage degenerative hip disease and who have failed non-operative management [1-3]. Hip resurfacing arthroplasty may offer greater retention of the biomechanical characteristics of a normal hip joint, lower dislocation rates, higher femoral bone preservation, and potentially easier revision surgeries in young active patients who may outlive their contemporary prosthetic device [4-6]. However, some authors believe that many of these advantages of resurfacing hip arthroplasty can be achieved in a standard primary total hip arthroplasty with the use of larger femoral heads [7].

At short- to mid-term follow-up, clinical success has been reported to be greater than 94% of patients who have undergone a resurfacing hip arthroplasty [8-11]. Clinical outcomes may improve when orthopaedic surgeons move beyond their learning curves and use narrower, more rigorous selection criteria [12,13]. With improving hip resurfacing results, a number of studies have compared the outcomes of standard primary total hip arthroplasty to resurfacing hip arthroplasty [1,14-20]. These studies have reported similar to superior post-operative activity levels, functional scores, and survival rates in the resurfacing patients. However, there is still disagreement about whether such findings are truly benefits of the procedure itself or reflect the higher pre-operative activity levels in patients treated with the resurfacing arthroplasty. Studies that can compare functional results of resurfacing hip arthroplasty to standard total hip arthroplasty across different patient populations and activity levels can give surgeons the further ability to make informed and patient-based decisions regarding their prosthesis selection [15,17-19,21-27].

Due to controversy over potential advantages or superiority of clinical outcomes of either of these arthroplasty procedures, this study examined the outcomes of patients who had undergone a resurfacing hip arthroplasty compared to all standard total hip arthroplasty procedures performed during the same period of time. For both groups, we assessed the following: (1) implant survivorship; (2) clinical outcomes; (3) activity scores; (4) complication rates, and (5) radiographic outcomes.

## Methods

**Figure 1:**
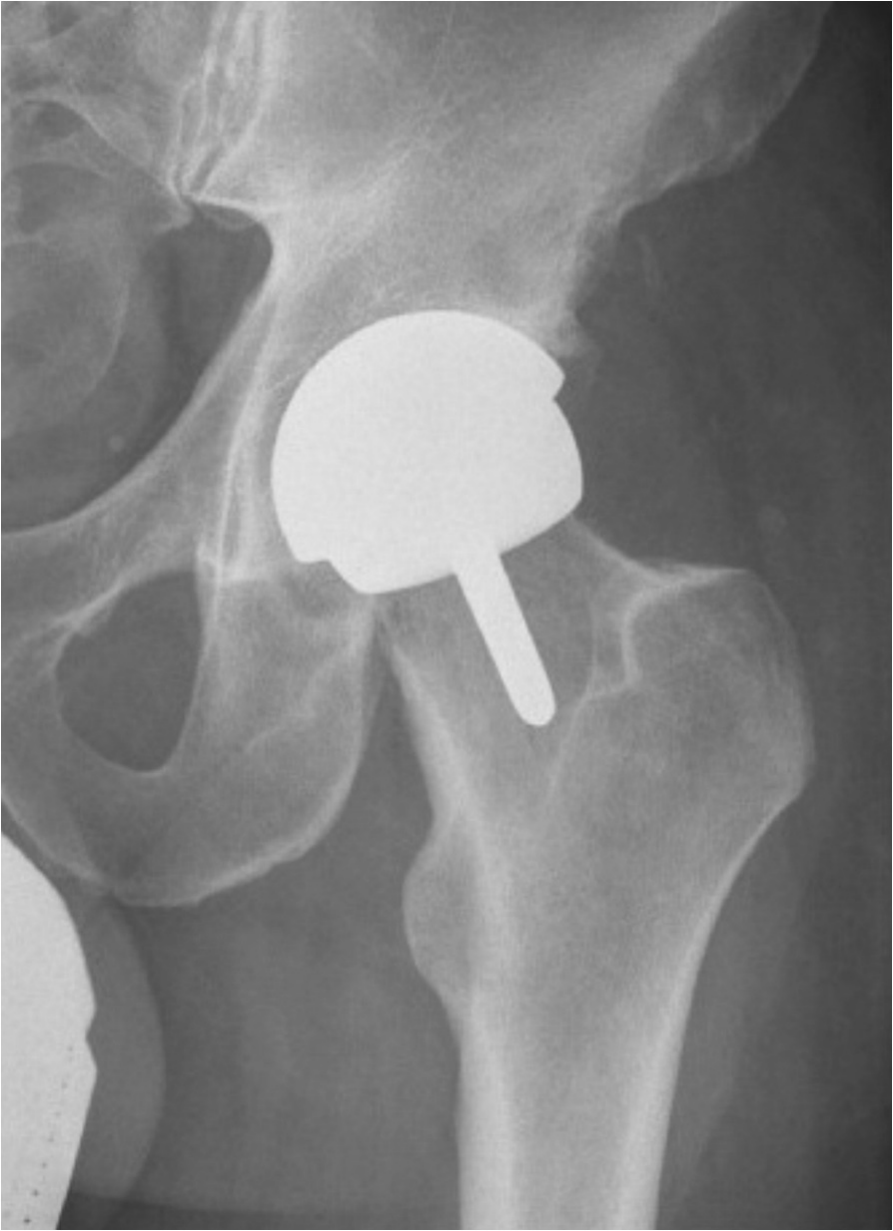
**Antero**-**posterior pelvis views of a patient who underwent a hip resurfacing arthroplasty using Cormet 2000**™ **(Corin**, **Cirencester**, **UK) prostheses.**

**Figure 2:**
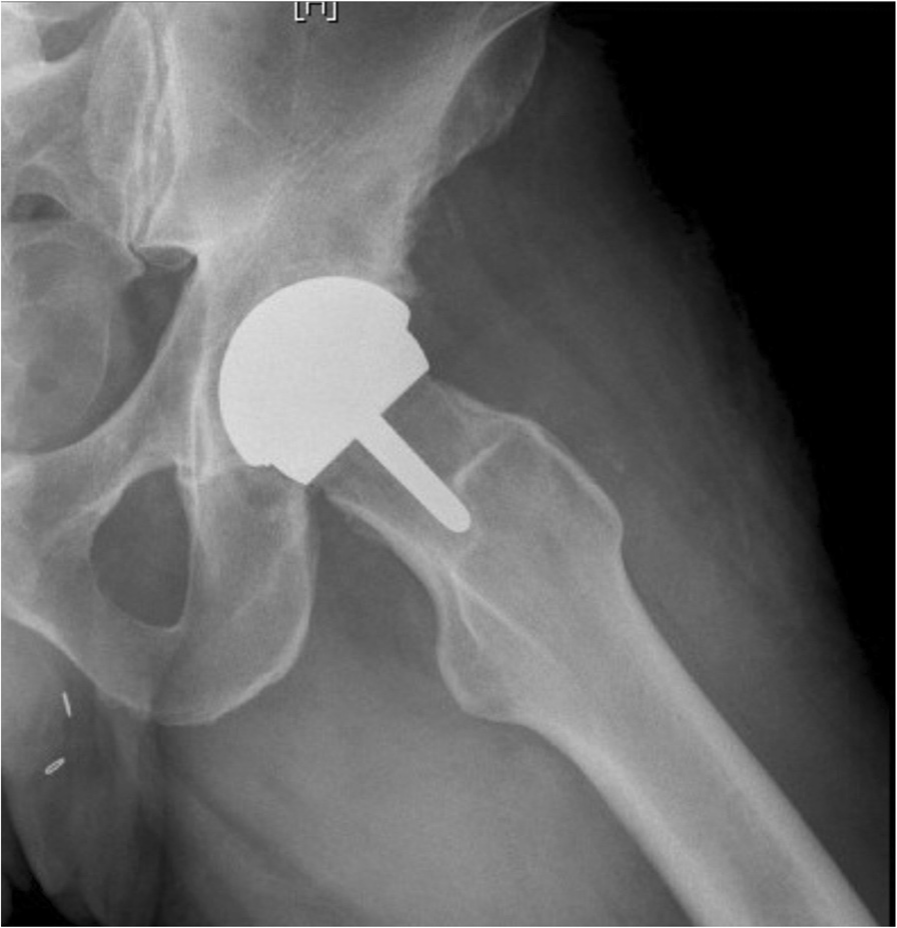
**Lateral frog pelvis views of a patient who underwent a hip resurfacing arthroplasty using Cormet 2000**™ **(Corin**, **Cirencester**, **UK) prostheses.**

A database of all consecutive patients who underwent a resurfacing hip arthroplasty using a new resurfacing system (Cormet 2000™, Corin, Cirencester, United Kingdom) (Figure 1 and 2) between November of 2007 and September of 2009 was reviewed. All procedures were performed at a single high-volume institution by an experienced fellowship trained adult reconstructive surgeon (MAM) who had performed over 1,000 prior resurfacing arthroplasty procedures using a different type of hip resurfacing system. The senior author switched to this new resurfacing system during November of 2007. This report includes all consecutive patients who had received the new resurfacing system and had a minimum of 24 months follow-up. One hundred and thirty one patients who had undergone 137 resurfacing arthroplasties were identified. All women were excluded from this study (6 hips) to reduce the gender bias from the study. One patient had been deceased due to metastatic cancer (with an intact prosthesis) and 10 patients (10 hips) were lost to follow-up (7%) prior to their 24-months post-operative visit and consequently were excluded from this study; although they had well-functioning prosthesis at last-follow-up. The remaining patients included 114 men (total of 120 hips) who had a mean age of 50 years (range, 20 to 85 years) at the time of their index procedure arthroplasty. All patients were evaluated both clinically and radiographically at a mean follow-up of 40 months (range, 24 to 55 months). All available medical records, including admission history and physical exams, pre-operative studies, post-operative reports, radiographs discharge summaries, as well as office notes were reviewed. A portion of the data from patients in the study and comparison groups at an earlier follow-up period has been previously published [21,28]. This study includes all these patients at a longer follow-up period as well as newly added consecutive patients.

Appropriate ethical approval for this study was obtained from the Institutional Review Board at Sinai Hospital of Baltimore for the study of these patients.

These patients were clinically compared to a consecutive cohort of men who had undergone a primary total hip arthroplasty during the same time period by the same surgeon. In this cohort there were 117 men who had a mean age of 53 years (range, 14 to 89 years) and a mean follow-up of 40 months (range, 24 to 55 months). There were no significant differences between age, gender, height, weight, body mass index, mean follow-up, and etiology of end-stage arthritis between the two cohorts (p= 0.09 to 0.48) (Demographic data for both groups are summarized Table 1).

**Table 1:** Demographics of the patients in both cohorts

	**Hip resurfacing cohort**	**Standard total hip arthroplasty**	***P*-value**
Number of patients	114	117	-
Number of hips	120	120	-
Gender	All men	All men	-
Mean age in years (range)	50 (20 to 85)	53 (18–78)	0.11
Mean body mass index in Kg/m^2^(range)	28.2 (19–45)	28.7 (19–51)	0.48
Mean follow-up in months (range)	42 (24–55)	40 (24 to 58)	0.23
Etiology of end-stage arthritis			
Osteoarthritis	111	106	
Osteonecrosis	9	14	0.38

All patients in our institution receive comprehensive pre-operative information regarding their optimal choice for undergoing resurfacing arthroplasty versus total hip arthroplasty. The senior author follows a narrow indication (young males who have femoral head sizes greater than 50 millimeters) to offer resurfacing procedure to the patients. However, the final choice is always made by the patients. All patients who had received total hip arthroplasty were either not an ideal patient based on senior author’s recommendation or had refused to receive a resurfacing arthroplasty based on their personal preferences.

All primary total hip arthroplasties were performed through an antero-lateral approach. All patients received an Accolade™ femoral component prosthesis (Stryker Orthopaedics, Mahwah, New Jersey) with a Trident ™ acetabular component (Stryker Orthopaedics, Mahwah, New Jersey). The acetabular prostheses were porous-coated and were implanted using a press-fit technique with or without screws. The femoral prosthetic component was a proximally porous-coated stem with a modular head, and was implanted using a press-fit technique.

All patients returned for follow-up visits at approximately six weeks, three months, six months, twelve months, and then yearly thereafter. At each follow-up visit, patients were examined thoroughly; clinical outcomes were further assessed using the Harris hip scoring system [29] and activity levels for all patients were determined a University of California Los Angeles (UCLA) activity scores pre- and post-operatively. Patients were also assessed for any medical or surgical complications such as prolonged wound drainage, hematoma formation, superficial or deep infection, deep venous thrombosis, or pulmonary embolism.

During each post-operative visit, antero-posterior and lateral views of the hips were obtained, and implants were evaluated radiographically for any periprosthetic fracture, progressive radiolucencies of the acetabular (DeLee and Charnley zones [30] or femoral component Gruen zones [31], implant subsidence, or component failure.

Revisions were defined as a change of the femoral or acetabular components for any reason including component loosening, peri-prosthetic fracture, osteolysis, component malalignment, or infection. Failure was defined as revision surgery due to any septic or aseptic reasons. Aseptic survivorship was defines as revision due to any aseptic failure.

All data were recorded using an Excel spreadsheet (Microsoft Corporation, Redmond, Washington) and all statistical data analyses were performed using SPSS software (Version 19, IBM Corporation, New York). Implant survivorship was evaluated and compared between the resurfacing and standard THA cohorts using Kaplan Meier analysis and Log-ranked test statistics. Harris hip scores and UCLA activity scores were compared using Mann–Whitney *U* test. Complication rate were compared using odds ratio statistics. A p-value of less than 0.05 was used as a threshold for significance.

## Results

The Kaplan-Meier implant survivorship in the hip resurfacing cohort was 98% (95% CI: 93.4 to 99.5%) at 36 and 48 months follow-up which were not significantly different than 99% (95% CI: 94.1 to 99.9%) in the standard total hip arthroplasty cohort (*p* = 0.95). In the resurfacing cohort, two patients (n= 2 out of 120) required a revision surgery: one for femoral neck fracture at 2 months and another for femoral head component loosening at 8 months after their index resurfacing arthroplasty procedure. The first patient underwent a successful revision surgery at our institution and achieved a Harris hip score of 92 points at 28 months follow-up. The other patient underwent a revision surgery at an outside institution and achieved Harris hip score of greater than 80 points. In the standard total hip arthroplasty group, there was 1 revision (n= 1 of 120) due to a peri-prosthetic fracture after a fall approximately 2 months after the index arthroplasty procedure. This patient was treated successfully with a femoral component revision and achieved a Harris hip score 82 at 36 months follow-up.

There were no significant differences in mean Harris hip scores between the hip resurfacing and patients total hip arthroplasty cohorts (*p* = 0.53). The mean Harris hip score in the hip resurfacing group improved from a mean of 47 points (range, 31 to 62 points) pre-operatively to a mean of 96 points (range, 72 to 100 points), post-operatively. The mean Harris hip score in the total hip arthroplasty cohort had improved from a mean of 41 points (range, 21 to 56 points) pre-operatively to a mean of 94 points (range, 70 to 100 points) post-operatively which was not significantly different from the hip resurfacing cohort (0.22) (clinical outcomes are summarized in Table 2).

**Table 2:** Summary of the clinical results

	**Metal-on-metal hip resurfacing**	**Standard total hip arthroplasty**	***P*-value**
Implant survivorship (%)	98	99	0.95
Complication rate (%)	0.8	0.8	1.0
Mean pre-operative Harris hip score (range)	47 (31–62)	41 (21–56)	0.75
Mean post-operative Harris hip score in points (range)	96 (72–100)	94 (70–100)	0.9
Pre-operative activity score in points (range)	3.6 (1–5)	2.7 (1–4)	0.001
Post-operative activity score in points (range)	6.7 (2–8)	5 (3–7)	0.001

There were significant differences in the mean post-operative UCLA activity scores between hip resurfacing and standard total hip arthroplasty cohorts (*p*=0.001). The mean pre-operative UCLA activity scores in the resurfacing cohort were 3.5 points (range, 2 to 5 points) which were significantly higher than 2.7 points (range, 1 to 4 points) in the standard THA cohort (p=0.005). Resurfacing patients also had achieved significantly higher mean post-operative (6.7 vs. 5 points; p=0.001) and mean gains in activity levels (3.1 vs. 2.3 points; p=0.002) compared to standard THA cohort.

There were no significant differences in complication rates between the two cohorts (p=1). In the resurfacing hip arthroplasty cohort, one patient developed a sciatic nerve palsy which was successfully treated with nerve decompression surgery. This patient regained full motor and sensory function and is doing well with achievement of a Harris hip score of 94 points at 24 months follow-up. In the standard total hip arthroplasty cohort, one patient developed a wound hematoma which was successfully treated by drainage. This patient is doing well and achieved a Harris hip score of 90 points at 30 months follow-up.

Except for the patients who had undergone a revision surgery for aseptic component failure, post-operative radiographic evaluation and zonal analysis of the remaining patients in both cohorts demonstrated no progressive radiolucencies, component malalignment, change in component position, or implant subsidence at their most recent follow-up (Figures 1 and 2).

## Discussion

Metal-on-metal hip resurfacing systems offer an alternative treatment to standard total hip arthroplasty in patients who have end-stage degenerative hip disease. Potential advantages include lower dislocation rate, higher femoral bone preservation, and potentially easier revision surgery in active patients who may outlive their contemporary prosthetic device. However, there is still disagreement about whether such findings are truly benefits of the procedure itself or reflect on higher pre-operative activity levels in patients treated with hip resurfacing arthroplasty. Comparison studies between resurfacing hip arthroplasty and standard total hip arthroplasty can give surgeons further ability to make patient-based decisions regarding their prosthesis selection [21]. The purpose of this study was to assess if similar clinical and radiographic outcomes were found when modern design hip resurfacing arthroplasty was compared to standard total hip arthroplasty at short- to mid-term follow-ups.

There were a number of limitations in the present study. This study only compared outcomes of hip resurfacing compared to standard total hip arthroplasty in men. Patient satisfaction and broader quality of life measures (such as SF-12 or SF-36, etc.) were also not evaluated or compared between the cohorts. In addition, the data still represent short-to mid-term outcomes in both patient groups. Nevertheless, the authors believe that the results are valuable since only a few studies have compared clinical outcome and activity levels of hip resurfacing arthroplasty compared to standard primary total hip arthroplasty.

Results of our study are in agreement with previous reports showing comparable clinical results of hip resurfacing to standard total hip arthroplasty. Pollard et al. [13] compared clinical outcomes of a cohort of 54 resurfacing patients (54 hips) matched to a cohort of standard total hip arthroplasties. At a mean follow-up of 61 months (range, 51 to 71 months) and 80 months (range, 42 to 120 months), 94% and 92% survivorship was reported in the hip resurfacing and standard total hip arthroplasty cohorts, respectively. Mont et al. [1] compared the clinical and radiographic outcomes of resurfacing arthroplasty and standard total hip arthroplasty in two closely matched groups of 54 patients who had a mean age of 55 years (range 35 to 79 years). At a mean follow-up of 40 months (range, 24 to 60 months), similar post-operative mean Harris hip score, revision rates, complications, pain score, and satisfaction ratings were reported for both groups; however, the resurfacing arthroplasty group had a significantly higher post-operative activity score (p<0.0001). Vail et al. [10] compared results of hip resurfacings in 52 patients (57 hips) compared to a cohort of 84 patients (93 hips) in a standard hip arthroplasty cohort. At a mean follow-up of 36 months (range, 24 to 48 months), they reported a 97% (n= 55 out of 57) and 96% (n= 89 out of 93) implant survivorship in the resurfacing and standard hip arthroplasty cohorts, respectively. Zywiel et al. [20] compared the clinical and radiographic outcomes of resurfacing arthroplasty and standard total hip arthroplasty in two matched groups of 33 patients who had a mean age of 53 years (range 37 to 79 years) and similar pre-operative activity scores. At a mean follow-up of 42 months (range, 25 to 68 months) significantly higher post-operative weighted activity scores were reported in the resurfacing group (p < 0.001); however, post-operative Harris hip score, revisions, satisfaction score, and pain score were similar in both groups. Our results are also in agreement with a previous report on the excellent results of Cormet 2000™ resurfacing system. Gross et al. [7] reported results of 373 Cromet 2000™ resurfacing arthroplasties at a mean follow-up of 8 years (range, 6 to 11 years). They reported a 93% implant survivorship when revision for any reason was used and 91% survivorship when radiographic failures were included.

## Conclusion

In summary, we have found a 98% implant survivorship at a mean of 42 months follow-up for patients who had undergone a resurfacing hip arthroplasty. Our results suggest that metal-on-metal hip resurfacing had similar overall outcomes when compared to standard total hip arthroplasty in terms of implant survivorship, clinical results, as well as complication rates. However, patients in the resurfacing cohort had achieved a significantly higher post-operative UCLA activity score. Overall, these findings may be contributed to the combination of patient selection as well as the prosthetic design. This is evident in higher pre-operative activity scores of the resurfacing group which can be attributed to patient selection, as well as, higher overall gains in activity scores in this group which can be related to the prosthetic design. The authors believe when patients meet the appropriate selection criteria, in the hands of experienced and high-volume arthroplasty surgeons, hip resurfacing provides excellent results at short- to mid-term follow-up. Further analysis to confirm such findings at a longer follow-up will be necessary.

## Competing interests

MAM receives royalties from Stryker; Wright Medical Technology, Inc.; is a consultant for Biocomposites; DJ Orthopaedics; Janssen; Joint Active Systems; Medtronic; Sage Products, Inc.; Stryker; TissueGene; Wright Medical Technology, Inc.; rDJ Orthopaedics; Joint Active Systems; National Institutes of Health (NIAMS & NICHD); Sage Products, Inc.; Stryker; Tissue Gene; Wright Medical Technology, Inc.; is on the board or editorial of American Journal of Orthopedics; Journal of Arthroplasty; Journal of Bone and Joint Surgery - American; Journal of Knee Surgery; Surgical Techniques International. BHK is a consultant for Sage Products Inc. Remaining authors have no competing interests. 

## Authors’ contributions

AP and QN have made substantial contributions to acquisition of data. KI, TT, and BHK have made substantial contributions to data analysis, interpretation of data, and preparation of manuscript. MAM has contributed all the patients, has made substantial contributions to data analysis, interpretation of data, manuscript preparation and revisions, as well as, final approval for submission. All authors read and approved the final manuscript.

## Financial disclosure

MAM receives royalties from Stryker; is a consultant for Janssen, Sage Products, Inc., Salient Surgical, Stryker, OCSI, TissueGene; receives institutional support from Stryker; and is on the Speakers Bureau for Sage Products, Inc. BK is on Speakers Bureau for Sage Products, Inc Remaining authors have no disclosures. Institutional support was received in support of this study.

## Pre-publication history

The pre-publication history for this paper can be accessed here:

http://www.biomedcentral.com/1471-2474/14/161/prepub
